# Ultrastructure of the Intramandibular Gland of Workers and Queens of the Stingless Bee, *Melipona quadrifasciata*

**DOI:** 10.1673/031.011.10701

**Published:** 2011-08-24

**Authors:** Carminda Da Cruz-Landim, Luciana F. Gracioli-Vitti, Fábio C. Abdalla

**Affiliations:** ^1^ Departamento de Biologia, Institute de Biociências de Rio Claro, Universidade Estadual Paulista (UNESP), 13506900 Rio Claro, Brazil; ^2^ Departamento de Biologia, Institute de Biociências de Rio Claro, Universidade Estadual Paulista (UNESP), 13506900 Rio Claro, Brazil; ^3^ Laboratório de Biologia Estrutural e Funcional, Universidade Federal de São Carlos (UFSCAR), Campus de Sorocaba, 18052-780 Sorocaba, Brazil

**Keywords:** exocrine glands, light microscopy, transmission electron microscopy, morphology, workers phases

## Abstract

The intramandibular glands of workers and queens of *Melipona quadrifasciata* Lepeletier (Hymenoptera: Apidae), at different ages and from different functional groups, were studied using light and transmission electron microscopy. The results demonstrated that these glands are composed of two types of secretory structures: 1.A hypertrophied epidermis on the dorsal side of the mandible that is an epithelial gland. 2. Free secretory cells filling the inner spaces of the appendices that constitute a unicellular gland. The epithelial gland is larger in the young (1-2-day-old workers), and the gland becomes involuted during the nurse worker stage. The unicellular glands of the workers posses some secretion during all of the studied phases, but secretory activity is more intensive in the foraging workers. Vesicles of secretion are absent in the unicellular glands of queens. These results demonstrate that these glands show functional adaptations in different castes corresponding to the functions of each caste.

## Introduction

Two types of mandibular glands are found in all adult hymenopterans. One type consists of a bag-shaped extra mandibular gland, which is known as the ecto-mandibular, or type I mandibular gland, and the other consists of free glandular cells of the mandible, known as the mesomandibular, or type II mandibular gland (Spradbery 1973; Quennedey 1998). Nedel (1960) first described the type II mandibular glands in *Apis mellifera* as secretory cells that occur in isolation, within the mandible and just below the epidermis. Additionally, Cruz-Landim (1967) observed that the epidermis of the mandible appeared hypertrophied in some species of stingless bees, which suggests a secretory function for this tissue. In a study of 11 species of stingless bees, Costa-Leonardo (1978) found that the intramandibular gland in workers consisted of two kinds of glandular structures: secretory cells termed class III gland cells that are individually provided with canals for secretion release, and a hypertrophied epidermis, termed class I gland cells according to the classification of Noirot and Quennedey (1991). Therefore, the intramandibular glands seem to be formed by two kinds of different secretory structures, the secretory cells that are freely distributed in the inner mandibular space and the hypertrophied epidermis that is located on the dorsal side of the mandible.

The intramandibular glands are epidermal glands that differentiate during metamorphosis and are most likely present in both sexes of all bee species (Salles and Gracioli 2002). Nevertheless, these glands have received little attention in previous investigations. Additionally, the chemical nature of the produced secretion and the secretory function are unknown.

The secretion of the intra-mandibular glands in Bombus (Hymenoptera: Apidae) is oleaginous, that Nedel (1960) suggests has an involvement in nest building (wax softening) or as a lubricator of the mandibles. In contrast, due to similarities between the class III cells from the intra-mandibular and ecto-mandibular glands, Costa-Leonardo (1978) proposed that these cells have the same function, perhaps for communication via pheromone production.

Indirect information about the possible function of these glands might be obtained by comparing changes in cell morphology and development among the different castes of individuals that compose the social colonies or the putative functional changes that occur during social interactions between them.

Santos et al. (2009) determined that differences existed among the newly emerged and the forager workers of *Plebeia emerina*, which were 20–30 days old. The hypertrophied epidermis was only present in forager workers.

As the intra-mandibular or meso-mandibular glands have not been researched as extensively as the other exocrine glands of bees, this work presents a morphological study of these glands from *Melipona quadrifasciata* Lepeletier (Hymenoptera: Apidae: Meliponini), which is a native stingless bee from Brazil.

## Materials and Methods

### Dissection and sample preparing

Four workers categories were collected from a colony of *M. quadrifasciata*: 1) during emergence from the comb cell, 2) when 1 to 2 days old, 3) when provisioning the brood cells (nurse workers) and 4) when returning from foraging (forager workers). In addition, virgin and physogastric queens were also collected.

### Light microscopy

Mandibles were excised from these individuals and fixed in 4% paraformaldehyde in 0.1M sodium phosphate buffer, pH 7.4. Then, the mandibles were embedded in resin JB - 4 (Polysciences, www.polysciences.com) according to the kit recommendations. The resin was polymerized at room temperature, and the blocks were cut into 6µm thick slices. The sections were put on histological slides and stained with hematoxylin and eosin (HE).

### Transmission electron microscopy

The mandibles excised from the bee specimens were fixed in 2.5% glutaradehyde in 0.1M cacodylate buffer, pH 7.2, overnight under refrigeration. After washing with cacodylate buffer, the mandibles were post-fixed in 1% osmium tetroxyde in the same buffer. Dehydration was performed in an acetone series, and then embedded in a blend of resin araldite (1.95 g) (Electron Microscopy Sciences, www.emsdiasum.com), LADD LX112 resin (2.05 g) (Research Industries Inc., www.laddresearch.com) and DDSA (3.32 g) (SPI Supplies Division of Structure Probe Inc., http://www.2spi.com) polymerized at 60° C and cut into sections. Sections were double stained with uranyl acetate and lead citrate, after which they were examined using a transmission electron microscope (C-100 Philips Electron Optics, Eindhoven, The Netherlands).

## Results

**Figure 1.**
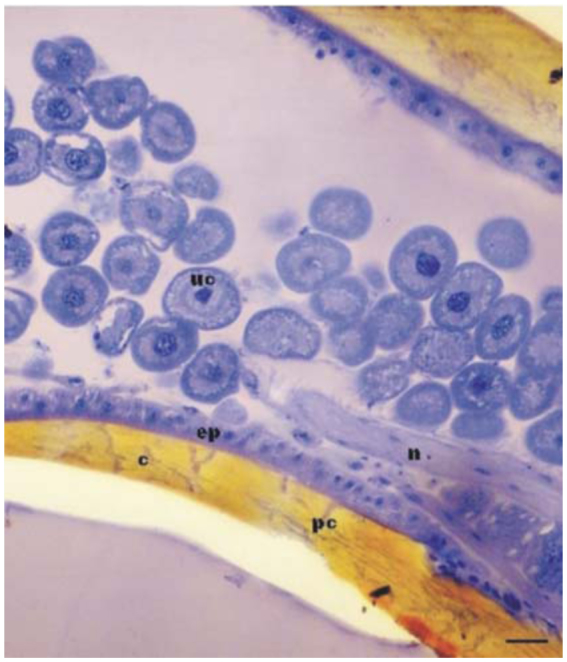
Light micrograph of a longitudinal section of a worker of *Melipona quadrifasciata* mandible showing the epithelial (ep) and unicellular (uc) glands. Note the pore canals (pc) in the cuticle (c). n= nerve. Bar= 40 µm. High quality figures are available online.

The mandibles of the studied individuals of *M. quadrifasciata* contain two types of secretory structures: a glandular epidermis in the dorsal side of the appendage that constitutes a type I or epithelial gland, and free secretory cells that fill the inner space and constitute a class III or unicellular gland, herein termed the intra-mandibular glands. The cuticle over the epithelial gland had many pore canals (Figure 1). The morphology of these two gland types exhibited marked changes in the different life phases of the individual and between different castes.

### Epithelial Gland

In adults, the epidermis consisted of an epithelium of flat cells. However, the epidermis may exhibit hypertrophy in certain places and form localized glands with variable functions. In workers and queens of *M. quadrifasciata*, the epidermis underlying the dorsal cuticle of mandibles is hypertrophied in newly emerged individuals.

### Workers

**Figure 2.**
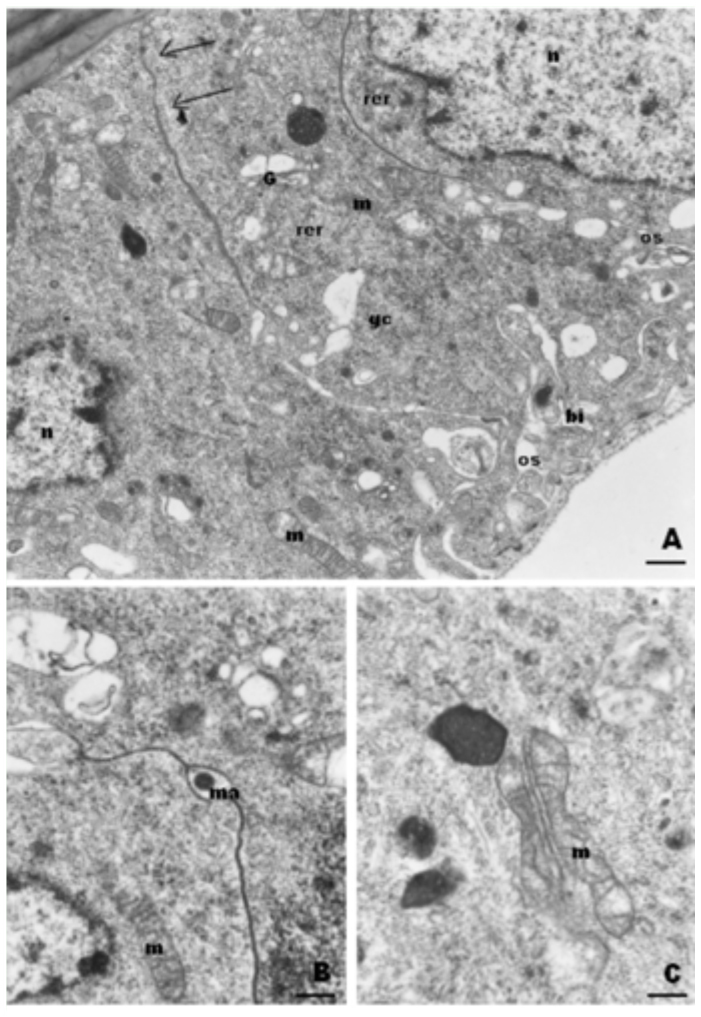
TEM micrographs from epithelial gland cells of newly emerged workers of *Melipona quadrifasciata*. A. Gland cells (gc) showing basal infoldings (bi), open basal intercellular spaces (os), and close apical ones (arrows). Bar= 0.7µm. B. Material (ma) circulating in the intercellular space. Note the septate desmosomes. Bar= 1 µm. C. Association between mitochondria and cell membrane. Bar= 1.5µm. m=mitochondria; rer= rough endoplasmic reticulum; G=Golgi; n=nucleus. High quality figures are available online.

**Figure 3.**
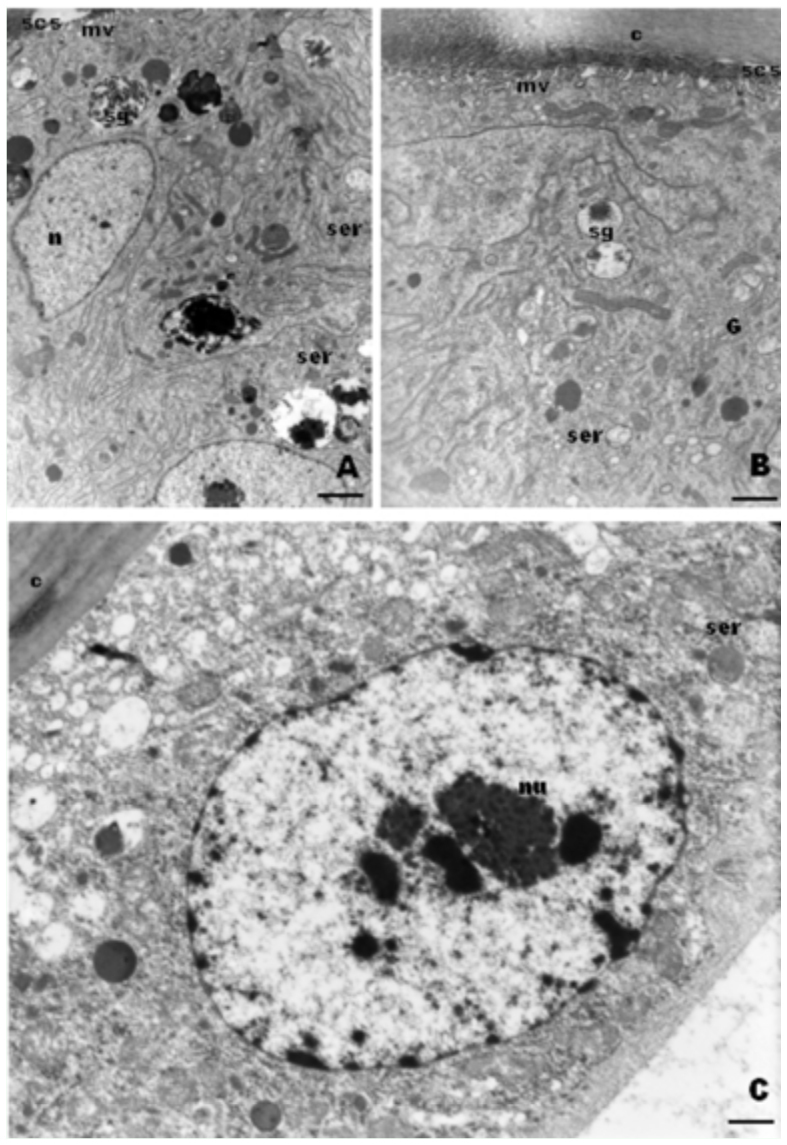
A, B. TEM micrographs of young workers of *Melipona quadrifasciata* showing granules with electrondense content (sg) and smooth endoplasmic reticulum (ser). Bar= 0.7µm. Note in B the apical microvillus-like (mv) projections and subcuticular space (scs). Bar= 0.7µm C. TEM of a gland cell from a nurse worker. Note the absence of granules and the basal lamina (bl) thickness. Bar= 0.4µm. n=nucleus; nu=nucleolus; G=Golgi; c=cuticle. High quality figures are available online.

1. In newly emerged workers, TEM examination revealed a single layered epithelium of cylindrical cells that had a folded basal plasma membrane and intercellular spaces that were open to the hemocoel but closed at the outer cuticle contact (Figure 2A). Material circulating through these open intercellular spaces was visualized (Figure 2B). The cells presented few profiles of RER, but many clusters of polyribosomes, and were rich in mitochondria, that in some cases, were associated with the intercellular plasma membranes (Figure 2C). The Golgi apparatus was present, but only a few electron-dense granules were observed, which could be secretions (Figure 2A).

2. In young workers, 1 to 2 days old, the cells developed several apical short microvillus-like projections whose top was linked to the cuticle by electron-dense reinforcements similar to focal contacts. A small sub-cuticular space developed among these microvilli. In addition, the basal plasma membrane was infolded, increasing in number and extension (Figures 3A and 3B). The RER profiles almost disappeared, and instead a SER and well-developed Golgi apparatus was observed as well as irregular granules that contain electron-dense material (Figure 3A).

3. The epithelial gland from nurse workers exhibited lower cells than the aforementioned young workers. The epithelium basal lamina thickened, and the plasma membrane basal and apical folds disappeared as well the electron-dense granules (Figure 3C).

4. In forager workers, the glandular epithelium almost returned to the condition of an undifferentiated epidermis.

### Queens

**Figure 4.**
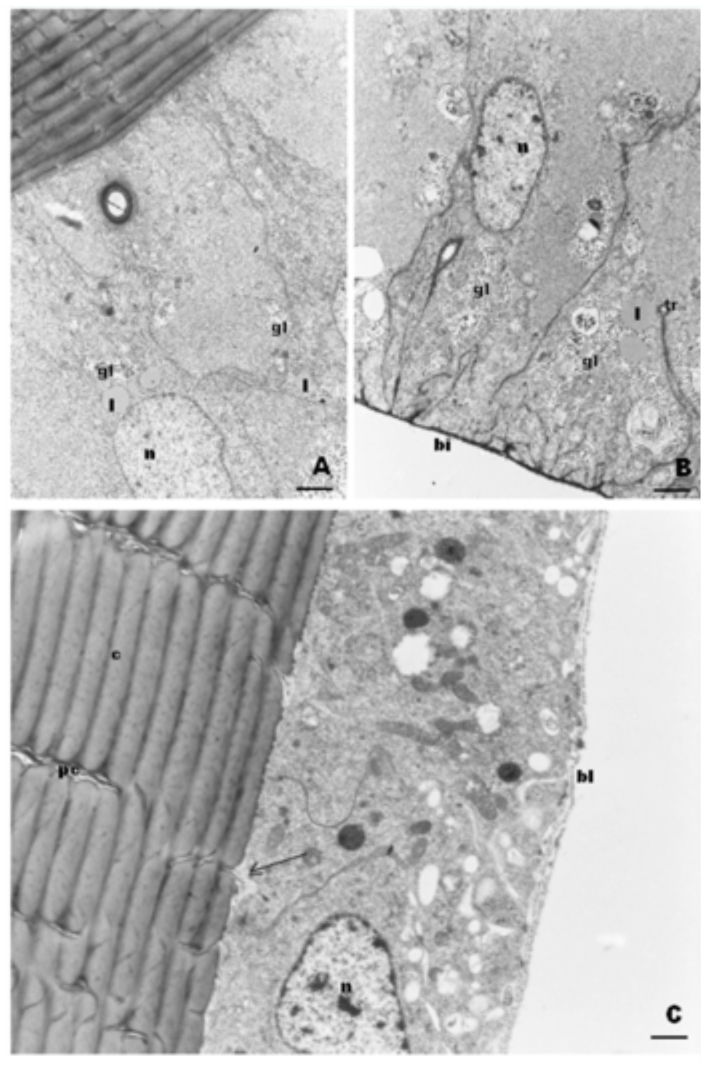
TEM of epithelial gland cells from *Melipona quadrifasciata* queens. A. In the apical region of cells from a virgin queen, there are few organelles and deposits of glycogen (gl) and lipid droplets (I). Bar= 0.8µm B. In the virgin queen, there are gland cell basal infoldings (bi), some containing tracheoles (tr) and glycogen deposits (gl). Bar= 0.5µm C. Epithelial gland from a physogastric queen showing pore canals (pc) in the cuticle (c) and material accumulated in the subcuticular space being eliminated through the canals (arrows), bl= basal lamina; n=nucleus. Bar= 0.4µm. High quality figures are available online.

In virgin queens, the cells of the epithelial gland were taller than in newly emerged workers. The cells presented many plasma membrane basal infoldings with the narrow lumen filled by electron dense material similar to the basal lamina. Some infoldings penetrated deep in the cell and had tracheolar branches. Nevertheless, the cellular contacts were closed. The cytoplasm was almost devoid of organelles, but numerous deposits of glycogen and some lipid droplets were present (Figures 4A and 4B).

**Figure 5.**
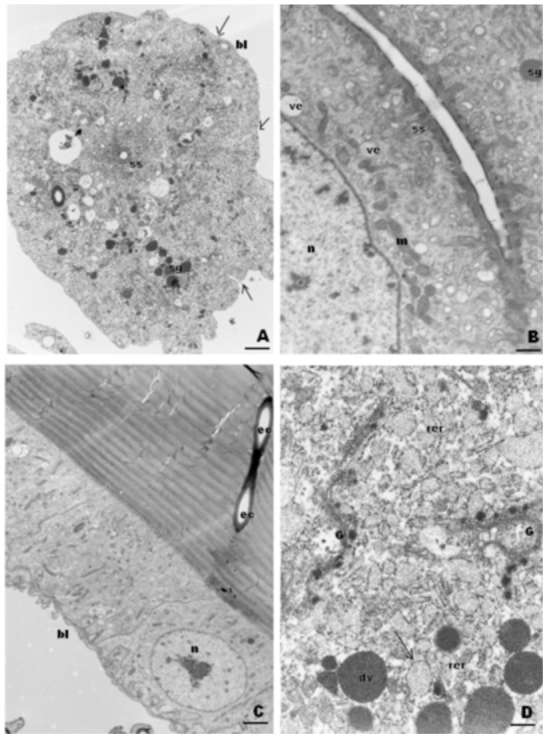
TEM of unicellular gland cells of workers of *Melipona quadrifasciata* A. Cell of a newly emerged worker showing peripheral folds (arrows) of a plasma membrane filled with material from basal lamina (bl), besides granules (sg) and secretor space (ss). Bar= 0.2µm B. Secretor space (ss) in the gland cell from a young worker. Bar= 0.8µm C. Excretory canals (ec) from the unicellular gland cells. Bar= 0.4µm D. Cytoplasm from a nurse worker cell showing rough endoplasmic reticulum (rer) storing secretion (arrows) and well-developed Golgi (G) with dense vesicles (dv). ve= transparent vesicles; Sg= secretor granules, m= mitochondria, n= nucleus. Bar= 1 µm. High quality figures are available online.

In physogastric queens, the epithelium was lower, and the plasma membrane basal infoldings and glycogen disappeared. Nevertheless, the cell had many mitochondria and free ribosomes (Figures 4C and 5C).

### Unicellular glands

The unicellular glands consisted of free spherical cells with a virtual intercellular secretory space lined by cuticle, which is the area to which secretion is delivered (Figures 5A and 5B). This space is connected to the excretory canal that is connected to the cuticle, through which the secretion is delivered to the outer surface of the integument (Figures 4A and 5C).

### Workers

In newly emerged workers, the gland cells had irregular contours and electron-dense granules in the cytoplasm, which most likely consist of secretion (Figure 5A). The plasma membrane forms short superficial infolds through which the basal lamina penetrates. The reticulum was predominantly rough and deposits of glycogen were generally present around dense granules.

A similar morphology was exhibited by the glands of young (1 to 2 days old) and nurse workers, but the number of mitochondria increased, and they appeared to be distributed primarily near the secretory space, that had numerous transparent vesicles (Figure 5B). The cells had RER cisternae with dilated lumen, which were filled with material of medium electron-density and well-developed Golgi apparatus consisting of lamellae and electron-dense small vesicles in addition to secretion granules (Figure 5D).

The gland cells of virgin and physogastric queens were very similar to those from nurse workers, but the electron-dense granules were absent. Only the vesicles seen around the secretory space were present. The gland cells from virgin queen had glycogen deposits (Figures 4A and 4B), which was a characteristic feature of cells from young individuals.

## Discussion

Intramandibular glands are found in all Hymenoptera. Billen and Espadaler (2002) described the epithelial gland in *Pyramica membranifera* and Amaral and Caetano (2005) found this gland in all castes of *Atta sexdens rubropilosa*.

The epithelial gland, as described by Noirot and Quennedey (1974; 1991) and Cruz-Landim (2002), discharges the secretion using the cuticle pore canals without another special cuticular structure. Glands of this type are relatively frequent in the epidermis of bees, but the better known are the wax glands. In this case, the gland cells have well-developed SER, which is typical of cells secreting lipid-like substances (Cruz-Landim 2000).

The present results indicate that the worker stage with the most developed epithelial intramandibular gland was the young workers (1 to 2 days old) and that in the nurse workers the gland begins involution. Although in newly emerged, nurse and forager workers, the RER seems to occur in young workers, but SER predominates. The epidermal cells of immature insects synthesize the cuticle, which in part consists of proteins, and cuticular layers continue to be deposited in some adult insects. The mandible is an important organ for the performance of workers several tasks, which include defense and foraging. The cuticle is poorly sclerotized in newly emerged workers, which suggest that the epidermis might still be secreting some of the cuticle compounds that would explain the presence of RER. The presence of SER is in accordance with the secretion of lipid-like material, which in the cells might be represented by the cytoplasm's electrondense granules, some with concentric lamination. Hydrocarbons secreted by some insect epidermal gland cells frequently present features similar to those of the electron-dense granules present in young workers (Hefetz and Orion 1982; Quennedey 1984; Percy-Cunningham and MacDonald, 1987; Tillman et al., 1999) suggesting the possibility of gland secretion contribution to surface hydrocarbons. Nevertheless, the permanence of the hypertrophy of the dorsal epidermis in young and nurse workers and the development of the RER suggests others specialized functions.

The unicellular glands of workers have granules and transparent vesicles that contain secretion in all stages studied, although the secretory activity appears to be more intense in foraging workers. Furthermore, the granules present in these glands are different from those seen in the epithelial glands, which are more compatible with protein content. Therefore, the epithelial and unicellular glands in these workers seem to have temporally different secretory cycles and different secretory products.

A putative function suggested for the epithelial gland in *M. quadrifasciata* is therefore, the production of cuticular hydrocarbons with higher activity when the young worker is acquiring its identity. However in *Plebeia emerina* both glands were more developed in foragers and the suggestion was made that the secretion of the epithelial gland was used in propolis manipulation (Santos et al. 2009).

However, our study indicates that the unicellular gland might be more useful for foraging workers. This type of gland is frequently found in several regions of the epidermis of bees (Cruz-Landim 1996; Cruz-Landim and Abdalla 2002; Guerino and Cruz-Landim 2002, 2003), and it almost always has characteristics of lipid-like producing glands, which was not observed in this study. This type of gland cell in insects is sometimes attributed to the secretion of the cuticle cement, but this component seems to be absent in bees (Chapman 1998). When the secretion delivering point is in articular membranes, the secretion is presumed to serve as a lubricant. Nevertheless, this type of gland is present in the abdomen of eusocial bees with marked dimorphism between castes (Cruz-Landim and Mota 1993; Cruz-Landim 1996; Cruz-Landim et al. 2005), and all indications suggest a pheromonal function. Nevertheless, the pheromones are frequently volatile, and the presence of RER in the cells of the unicellular gland, as well the morphology of the secretion granules, seems to be incompatible with secretion of volatile pheromones. However, the secretion of a contact, non-volatile pheromone by these glands cannot be excluded due to the frequent interactions among workers via mandibles. The gland cells have secretion granules in all stages of development, but their morphology indicates they are most active in the nurse workers.

Queen mandibles are not utilized in the same manner as those of workers are, and they are never as sclerotized as those of the worker. Furthermore, the apparent absence of a secretory function in the epithelial gland deserves more investigation. The epidermis hypertrophy in virgin queens might only reflect the youth of the queen, as indicated by the presence of glycogen deposits in unicellular gland cells. The electron-dense granules assumed as secretion in workers are absent from the glands of the virgin and physogastric queens. Instead, electron-transparent vesicles are located around the intercellular secretory space, which demonstrates that the secretion produced by this gland in queens is different from workers.

The unicellular glands exhibited more morphological variability during bee life. For both glands, the timing of higher activity was different. The most active phase of the glands appeared in newly emerged queens, while this occurred later in workers. These results demonstrate that the same glands from female individuals of same species might exhibit different functional adaptations, which allow for the specialized functions of each gland.

## Abbreviations

LM: light microscopy;
RER: rough endoplasm reticulum;
SER: smooth endoplasm reticulum;
TEM: transmission electron microscopy
